# The Limitations on Vergence Responses to Naturalistic Images Resulting From Simulated Central Scotomas and Defocused Peripheral Retina

**DOI:** 10.1007/s44402-026-00028-0

**Published:** 2026-03-06

**Authors:** Bhagya L. Marella, Sonisha Neupane, Clara Mestre, T. Rowan Candy

**Affiliations:** 1 School of Optometry, Indiana University, Bloomington, Indiana USA; 2 Present Address: Center for Sensors, Instruments and Systems Development, Universitat Politècnica de Catalunya, Barcelona, Spain

**Keywords:** Anisometropia, Disparity, Refractive error, Scotoma, Suppression, Vergence

## Abstract

**Purpose:**

Clinical visual disorders are associated with interocular suppression and scotomas of the central visual field. The goal of this study was to understand the ability of the peripheral retina to drive reflex vergence responses in the presence of refractive defocus.

**Methods:**

Vergence responses to steps in horizontal disparity were recorded from functionally emmetropic adults (15–51 years of age) viewing grayscale outdoor natural images. Mean luminance central scotomas of 0°–25° radius in 5° steps were simulated in Experiment 1. In a second experiment, bilateral (1.50 D and 3.00 D) or unilateral (0, 0.75 D, 1.50 D, 2.25 D and 3.00 D) defocus, computed for each participant’s pupil size, was simulated with scotoma radii of 0°, 5°, 8° and 10°.

**Results:**

Vergence responses to these disparity steps demonstrated a stereotypical tuning function in the absence of scotomas or defocus. In Experiment 1, bilateral scotoma radii of 10° or more led to significant decreases in open-loop vergence response amplitude (*p* < 0.001 for all). In Experiment 2, there were significant reductions in open-loop vergence responses resulting from unilateral (*p* < 0.001) but not bilateral defocus (*p* = 0.93). The effect of radius was significant, with no effect of bilateral versus unilateral scotoma format.

**Conclusion:**

These data characterise the ability of the peripheral retina to maintain eye alignment for naturalistic images in the presence of central scotomas or suppression, and defocus. They suggest that vergence performance is reduced for natural images with bilateral and unilateral central scotomas, and indicate a need for refractive correction of anisometropia >1.50 D in order for the peripheral retina to support vergence function fully.

Key Points

- Vergence performance was reduced in the presence of both bilateral and unilateral scotomas with the reduction in disparity cues.
- Anisometropia of greater than 1.50 D needs to be corrected to support ocular alignment in the presence of both unilateral and bilateral scotomas.
- The shape of the disparity tuning functions was robust and consistent even at the larger scotoma sizes and higher defocus levels.

## Introduction

Interocular retinal disparity serves as the error cue providing feedback for aligning the eyes in a three-dimensional environment. Unfortunately, numerous clinical disorders disrupt the most sensitive central retina [1–6] and affected patients must rely on peripheral retinal regions with limited sampling and increased pooling of spatial information to provide the disparity cue [7–9]. How well can the visual system maintain eye alignment using fusional vergence responses in the absence of binocular function in the central retina, and how is this impacted by refractive error?

Patients with strabismus regularly exhibit suppression of information from the central retina of their deviated eye in binocular viewing conditions, presumably as an adaptation to avoid diplopia and confusion. Burian [8] and Winkelman [9] were the first to demonstrate fusional vergence responses to retinal disparity stimuli presented in the peripheral visual field of strabismic patients. Boman and Kertesz [10] showed that strabismic patients made fusional motor responses when the disparity stimuli were presented both across the full field and in the periphery alone, but not when the disparate stimuli were presented to the central retina only. Others have characterised the responses of typical individuals to images with simulated missing regions, i.e., scotomas. Kertesz and Hampton [11] demonstrated that a simulated 10° diameter monocular scotoma was sufficient to disrupt vergence responses of typical individuals to disparity stimuli, while Eizenman et al. [12] found that while vergence occurred for the tested monocular scotoma diameters of 2°–10° during the initial open-loop response phase without visual feedback, its gain decreased with increasing diameter. Yang et al. [13] also demonstrated a decrease in open-loop vergence response and an increase in vergence latency with simulated monocular scotomas. In addition to suppression scotomas resulting from atypical binocular vision, Verghese et al. [14], Vullings et al. [15] and Shanidze et al. [16] have shown reduced oculomotor responses (saccade amplitude and pursuit gain) for participants with bilateral and unilateral central scotomas resulting from macular degeneration.

Patients with scotomas must rely on retinal images of the natural environment to drive accurate eye alignment during their habitual daily activities, and clinicians must also decide whether to correct refractive error for these patients with limited foveal function. The current study was designed to expand upon these previous investigations to understand how the statistics of grayscale natural images can support eye alignment in the presence of both central scotomas and peripheral defocus. Symbols or lines were used as the peripheral targets to provide disparity cues in previous studies, and the boundaries of simulated scotomas were distinct [11, 12]. Such stimuli represent only a limited subset of the daily content experienced by these patients, and the typical borders of suppression or pathological scotomas are indistinct. Here, participants with typical vision were presented with large natural scene stimuli with combinations of computationally simulated unilateral and bilateral peripheral defocus and central scotomas. The scotomas were simulated using both the mean luminance of the natural image to create a loss of contrast in post-retinal neural processing, and a gradual transition zone to the peripheral image to mimic the central suppression or pathological scotomas reported by clinical patients [17, 18].

The central retina can resolve finer spatial information and is more sensitive to loss of contrast than the peripheral retina [19]. Secondly, to the extent that accommodation is consensual [20, 21], unilateral defocus cannot be overcome with accommodation, whereas bilateral defocus could be overcome by pre-presbyopic patients who are not myopic and thereby focused closer than the target. Both of these points suggest that anisometropic patients with unilateral scotomas would be at particular risk for inaccurate vergence. In the current study, computational simulation of defocus was used to understand the accurate spatial frequency-specific impact of loss of contrast [22] resulting from defocus, while the best focus was still in the plane of the screen (creating a stable accommodation demand and only loss of contrast at these spatial frequencies).

The hypotheses regarding the reflex vergence responses [23–26] of participants with typical vision to these stimuli were that: (i) the peripheral retina would not be capable of matching typical foveally-driven vergence performance, as demonstrated previously, (ii) the impact of refractive error on fusional vergence responses would reduce with increasing scotoma size, as a result of the reduction in spatial sensitivity in the peripheral retina, (iii) if there is an effect of refractive error in the peripheral retina, unilateral defocus would have a greater impact on fusional vergence performance than bilateral defocus (as demonstrated for binocular perception) [27–30].

## Materials and Methods

### Participants

Thirty-one participants (age: 15–51 years) with normal visual function who were naïve to the study protocol were recruited from the local students and staff. All participants had been through a comprehensive clinical examination to determine their best refractive correction (subjective) and ensure typical stereoacuity (<50 arc seconds), binocular vision status (no atypical heterophoria) and near point of convergence (<10 cm). They had best corrected distance visual acuity better than 6/6 in each eye with no evidence of atypical conditions that would impact vergence performance. All completed the study visits successfully. The study was approved by the institutional review board of Indiana University and followed the tenets of the Declaration of Helsinki. Informed written consent was obtained from all participants.

### Equipment and Stimulus Presentation

**Fig. 1:**
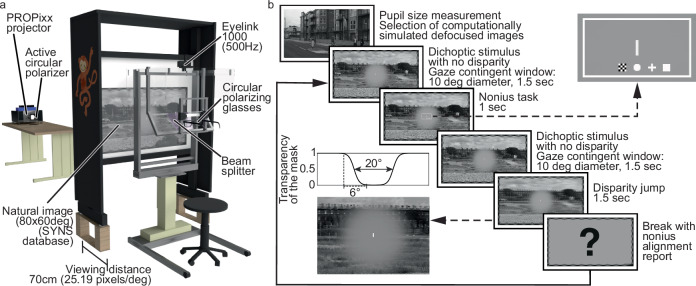
Experimental apparatus and trial design. **a** Design of the equipment. **b** The stages of each experimental trial. SYNS Southampton-York Natural Scenes [31].

Twenty-one different natural photographs of distant scenes were chosen from the Southampton-York Natural Scenes dataset (SYNS: syns.soton.ac.uk) [31]. These images needed to be converted to grayscale for dichoptic presentation using a PROPixx projector in RB3D mode (VPixx Technologies: vpixx.com). The images were adjusted to match each other in mean luminance to establish a stable pupil diameter for the defocus simulations. The resolution of the projector was 1920 and 1080 pixels horizontally and vertically, respectively. Presentation of the alternating right and left eye frames was synchronised with a VPixx DepthQ active circular polariser (frame rate: 120 Hz) to enable dichoptic presentation while the subject wore the appropriate polarising spectacles (Fig. 1a). The images were presented at a viewing distance of 70 cm. This viewing distance was chosen to make the screen as large as possible in angular units while avoiding the impact of a small change in viewing distance leading to a large dioptric or metre angle difference at very close distances. This middle distance also permitted the disparity tuning curves for both convergence and divergence demands to be determined. The rear projection screen maintained the circular polarisation (ScreenTech: screen-tech.eu/) while crosstalk between the two eyes’ polarisation states was minimised by subtraction of contrast using PROPixx RB3D mode image correction. Matlab® (version: 9.10.0 (R2021a), mathworks.com) and Psychtoolbox [32] were used for image presentation and defocus simulations (Indiana Retinal Image Simulator (IRIS) software) [33]. Eye movements were recorded binocularly at a sampling rate of 500 Hz using an Eyelink 1000 (SR-Research Ltd: sr-research.com/) mounted above the screen with a hot mirror beamsplitter (Fig. 1a). Compared with a table-top placement, this arrangement removed the Eyelink camera from the participant’s field of view to prevent it from occluding part of the stimulus image or acting as a fusion lock. The edge serration of the dichoptic images was in opposite phase for the two eyes, also to prevent the edge acting as a peripheral fusion lock, and the room lights were turned off during data collection to reduce spatial contrast at the frame around the screen.

Central scotomas were simulated by blending the natural image with a disc at the mean luminance of the image using Psychtoolbox (Fig. 1b). The edge of the disc was a logistic cumulative distribution function transition from opaque disc to full transparency. This approach mimicked changing the depth of the scotoma, transitioning from zero contrast in the scotoma to the full contrast peripheral natural image [17, 34]. The scotoma radius was defined as the distance from the centre of the scotoma to the 50% transparency point. Spherical defocus was simulated in the periphery by convolving each natural image with a point spread function computed for the appropriate defocus magnitude and the individual’s measured pupil diameter (IRIS software [33]). During the trials, the peripheral natural stimulus underwent a step change in disparity while the scotoma remained stationary. This approach mimics a natural peripheral disparity stimulus before any eye movement. A change in vergence alignment in response to this disparity stimulus would lead to a shift in the simulated scotoma position on each retina, whereas a real scotoma would move with the eye. The effect of this limitation was reduced by performing the primary analyses on the open-loop initial phase of the vergence response (a period of two latencies after the disparity change), before any visual feedback could affect the later components of the response [35–37].

### Procedure

Participants were positioned with a chin and forehead rest at a viewing distance of 70 cm from the screen (Fig. 1a). Thirteen-point calibration and validation routines were performed for the Eyelink recordings and only accepted if the validation deviation was less than 1° at the locations tested. Pupil size was measured using the Eyelink 1000 during four consecutive 1-s time windows, while participants maintained fixation in a 10° gaze-contingent window at the centre of one of the natural scene images. If the difference between the maximum and minimum values for the four pupil sizes recorded in each eye was >0.5 mm, then the measurement procedure was repeated. The mean of the right and left eye pupil sizes, rounded to the nearest 0.5 mm, was used to identify the appropriate simulated defocus images.

Participants were then presented with the dichoptic natural images in a series of conditions varying in scotoma size, defocus and disparity step size. Each trial began with the images presented at zero disparity (in the plane of the screen) with a 1° vertical fixation line presented only to the left eye (Fig. 1b). After the participants fixated the line to within the gaze-contingent window for 1.5 s, a set of four symbols spaced at 1.5° intervals was presented beneath the fixation line to the other eye for 1 s and the participant was asked to remember which symbol was aligned with the fixation line. The horizontal location of these symbols was changed randomly for each trial, but one was always aligned with the fixation line if the participant was appropriately converged to the plane of the screen. After this Nonius alignment task, the 1.5 s gaze-contingent window was repeated, and the images were stepped to a new disparity for 1.5 s [23, 24]. Participants were asked to maintain central fixation on the line presented monocularly during this interval. Finally, a blank screen was presented while the participant blinked, relaxed their eyes and reported the remembered symbol aligned with the fixation line using a RESPONSEPixx button box (VPixx Technologies: vpixx.com). This button press triggered the next trial.

### Stimulus Conditions

#### Experiment 1

First, a group of 17 participants were tested using bilateral scotoma radii of 0°, 5°, 10°, 15°, 20° and 25° to the centre of the transition zone. Crossed and uncrossed disparity steps in the image of 0°, 0.5°, 1°, 2°, 4° and 8° behind the static scotomas were presented in six repetitions of each combination. The size of natural images used in this experiment was 80° horizontally and 60° vertically, which filled approximately 70% of a typical human binocular horizontal visual field and 50% of the vertical field. The order of the 396 trials was randomised, and data were collected during two separate visits (66 trials/session; 3 sessions/visit).

#### Experiment 2

In the second experiment, 14 participants were recruited to determine the effect of unilateral and bilateral scotomas, as well as defocus magnitude and format, on the vergence responses. The size of the natural images used in this experiment was 76° × 43°. In experiment 1, larger images were used to simulate the larger scotoma size, while a smaller, higher-resolution display was used in experiment 2 to achieve more accurate defocus simulations. The first experiment revealed that radii larger than 10° were sufficient to reduce the vergence responses, so the second experiment sought to test whether defocus magnitude and format led to a reduction at scotoma sizes below 10°. The combinations of scotoma format and radius, defocus format and magnitude, and disparity step size were presented in randomised order. The scotoma radii were 0° (no scotoma), 5°, 8° and 10° in either unilateral or bilateral format. Spherical defocus values of 0, 0.75, 1.50, 2.25 and 3.00 D were presented unilaterally to each eye, and 1.50 and 3.00 D of defocus were presented bilaterally. Crossed and uncrossed disparities of 0°, 1.5° and 3° were presented for each scotoma and defocus combination and repeated four times, resulting in a total of 1568 trials. The trial order was randomised, and data were collected over eight sessions to avoid fatigue.

A set of control trials was also presented in the second experiment, to examine the alignment of the eyes under dissociated conditions (heterophoria): (i) Monocular stimulation: in order to mimic a unilateral cover test, images with bilateral scotomas of 2° or 8° were presented at zero disparity and then one eye’s image was changed to mean luminance mimicking occlusion of the eye, (ii) Different images: images with bilateral scotomas of either 2° or 8° radius were presented at zero disparity and then different natural images were presented to the two eyes to eliminate correspondence, (iii) Alternating cover test (ALT): a central fixation cross was presented alternately for 3 s to one eye and then the other, while the other eye was presented with the mean luminance of the image.

### Data Analysis

Horizontal and vertical eye movements of the right and left eyes, blinks and missed samples were computed for each trial. Linear interpolation was performed across blinks and periods of missing data. The eye movement data were then smoothed using a zero-phase finite impulse response low-pass filter with a cut-off frequency of 20 Hz. Trials were excluded if: (1) there was a blink during the response to the disparity step. (2) There was >1° change in version between −200 and +400 ms from the stimulus onset. (3) There was >1° change in vergence during a ±200 ms interval around the stimulus presentation (1 latency period). (4) Vergence acceleration was estimated to be greater than 500°/s^2^ during an interval of −200 to +400 ms around the stimulus presentation. Included trials were normalised to set the initial vergence position to zero, and vergence responses were averaged within each condition.

**Fig. 2:**
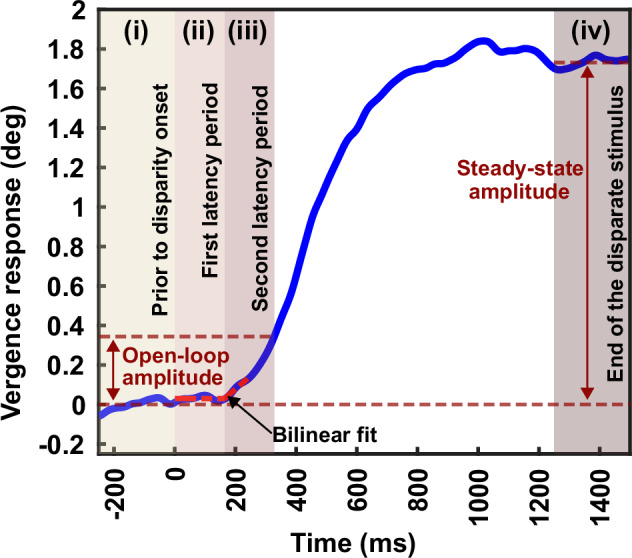
The vergence response (blue) collected during a single trial illustrating the different phases of the response: (i) the stable vergence alignment before the stimulus onset at time 0 ms, (ii) the estimated response latency, (iii) the open-loop response and (iv) the steady-state amplitude period. The dashed bright red line on the blue curve between 0 and 300 ms is the bilinear fit used to determine the response latency.

Summary analyses were performed on the open-loop vergence response amplitudes [37]. This response is defined as the period over which the vergence motor response was not influenced by visual feedback about the movement of the eyes [37]. It was computed as the change in vergence alignment between the onset of the stimulus and the end of the second latency period of the motor response (Fig. 2), the logic being that any retinal experience of the vergence response cannot be acted upon until a second latency period has passed. The open-loop period isolates the response to the original disparity step, and was not influenced by the fact that other cues for alignment (e.g., defocus, size cues and the lack of movement of the simulated scotomas) were now inconsistent with the change in eye alignment occurring in response to the disparity [37]. The latency period for the vergence response was determined by fitting a bilinear function (Fig. 2, red dashed line) over a 300 ms window after the stimulus onset. The *lsqnonlin* function in Matlab was used to estimate the location of the intersection of the two lines using nonlinear least squares optimisation, with an initial estimate of 150 ms for the latency, a slope in the direction of the disparity step and without constraining the intersection value to lie within a specific range of time [37]. The resulting fits were examined visually, and those with poor fits, or latencies that were negative or >300 ms were refitted to generate reliable estimates. The trial was excluded if that was not possible.

**Fig. 3:**
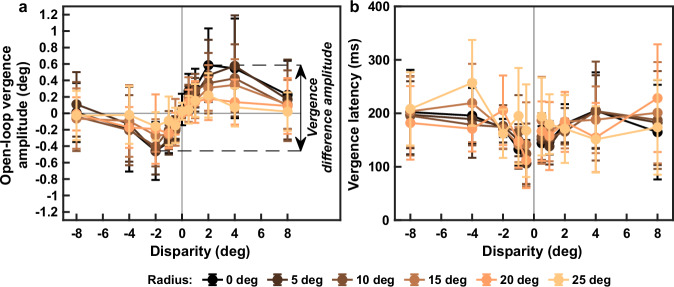
Experiment 1: The effect of bilateral scotoma radius on mean ± SD **a** open-loop amplitudes and **b** latency as a function of disparity step size. Positive and negative values on the *x*-axis correspond to crossed and uncrossed disparities, respectively. Similarly, positive and negative values on the *y*-axis represent convergence and divergence responses, respectively. The same sign convention is followed in all the disparity tuning function plots below. Deg degrees, SD standard deviation.

The difference in response amplitude between the convergent and divergent peak of each disparity tuning function was used as the final metric to interpret the effects of scotoma size and format, and defocus magnitude and format (Fig. 3a). Linear mixed models implemented in R (r-project.org) were used to summarise the effects of the experimental manipulations on this summary metric (Tables 1–3). Contrasts were computed for the post-hoc analysis, with Tukey adjustments for multiple comparisons.

### Ethics Approval

Approved by the Institutional Review Board of Indiana University.

## Results

All participants completed the Nonius task. Each reported the expected symbol, at a disparity of 0° for the plane of the screen, after more than 99% of trials across conditions. This confirmed that participants’ eyes were converged within approximately 1° of the plane of the screen before the step stimulus was presented. The mean (standard deviation) heterophoria position for this 70 cm viewing distance, determined from the ALT control condition for participants in experiment 2, was 0.39° of exophoria (0.50°), which is equivalent to <1Δ (prism dioptre).

### Experiment 1: Effect of Bilateral Scotoma Size

The first experiment determined the effect of bilateral scotoma radius on the vergence responses. The amplitude of the initial open-loop portion of the reflex response is summarised as a function of scotoma radius in Fig. 3a. These disparity tuning functions are consistent with the literature [22–24], indicating a typical peak response at approximately 2°–4° of disparity for both convergence and divergence (Fig. 3a) [13, 23, 24]. The peak-to-trough open-loop vergence difference amplitude was calculated as the difference in response between ±2° disparity (Fig. 3a). In a linear mixed effects model (*lmer*), this difference amplitude was predicted with radius as a categorical fixed factor, participant as a random intercept and a baseline model of no scotoma. A post-hoc contrast analysis revealed radii of 10°, 15°, 20° and 25° all resulted in difference amplitudes that were significantly smaller than the no scotoma condition (all *p* < 0.001). The analysis of latency included log magnitude of disparity as a continuous fixed factor, radius as a categorical fixed factor, the interaction between radius and disparity as a fixed factor and participant as a random intercept, with the peak vergence response of 2° disparity and no scotoma as the baseline model. There was a significant main effect of disparity magnitude on the response latencies (*p* = 0.004), but no significant effect of scotoma radius (*p* = 0.73). A post-hoc contrast analysis of log disparity magnitude showed that latencies at 0.5° and 1° disparities were significantly shorter, while those at 4° and 8° were significantly longer than for the 2° disparity condition (all *p* < 0.001). This relationship between latency and log disparity magnitude demonstrated a linear trend, with larger disparity resulting in longer latency. The interaction between radius and log disparity magnitude was also significant (*p* = 0.005), with the increase in latency with disparity being lost at the scotoma radius of 25°.

### Experiment 2: Effect of Scotoma Format for Radii up to 10°

**Fig. 4:**
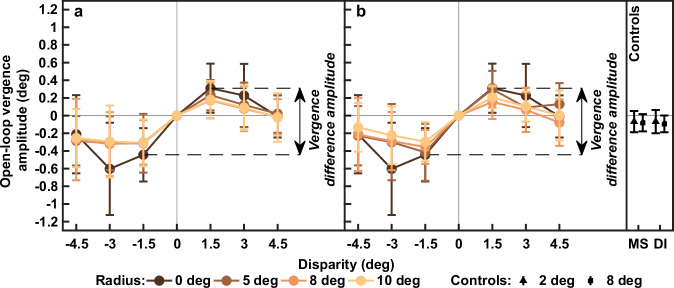
Experiment 2: Mean ± SD open-loop vergence responses plotted for the simulated **a** unilateral and **b** bilateral scotomas for the no defocus conditions. Positive and negative values correspond to convergence and divergence, respectively. Deg degrees, MS monocular stimulus, DI different images, SD standard deviation.

**Fig. 5:**
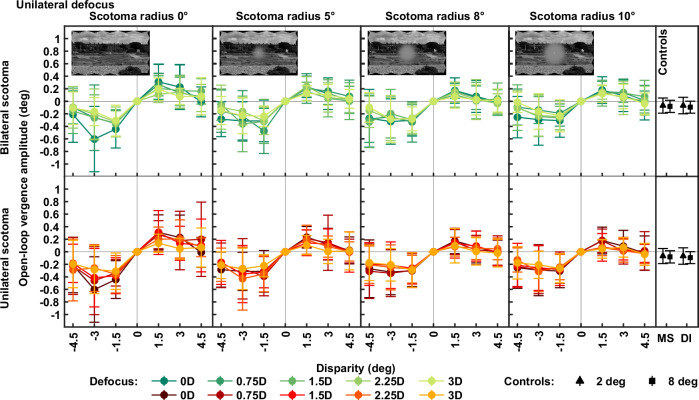
The effect of unilateral defocus magnitude and scotoma radius on mean ± SD open-loop vergence amplitudes: Top row: bilateral scotoma, Bottom row: unilateral scotoma. Positive values correspond to crossed disparity and convergence. The data from the control conditions are plotted again for comparison. Deg degrees, MS monocular stimulus, DI different images, SD standard deviation.

**Fig. 6:**
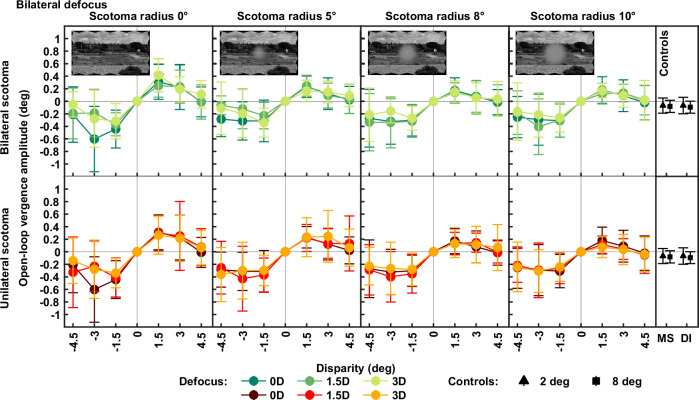
The effect of bilateral defocus magnitude and scotoma radius on mean ± SD open-loop vergence amplitudes: Top row: bilateral scotoma, Bottom row: unilateral scotoma. Positive values indicate crossed disparity and convergence. Deg degrees, MS monocular stimulus, DI different images, SD standard deviation.

In the second experiment, 14 participants were tested to determine the effect of scotoma format and defocus magnitude and format on vergence responses, with scotoma radii of 0°, 5°, 8° and 10°. The effect of scotoma format without defocus is shown in Fig. 4, and the effects of defocus and its format are shown in Figs. 5 and 6. The peak-to-trough vergence difference amplitude in the second experiment was computed as the difference in open-loop response between ±1.5° of disparity.

A linear mixed model analysis predicted the vergence difference amplitude for the no defocus unilateral and bilateral scotoma conditions (Fig. 4), keeping scotoma radius (continuous variable) and format (categorical variable) as fixed factors with their interaction, and participant as a random intercept. The peak-to-trough open-loop vergence difference amplitude decreased somewhat with an increase in scotoma radius (Table 1, *p* = 0.03). There was no main effect of scotoma format (Table 1, *p* = 0.14), indicating that unilateral and bilateral scotomas had a comparable impact for radii up to 10°, presumably because they both eliminated disparity cues for fusional vergence. The interaction between scotoma format and radius was also borderline significant (Table 1, *p* = 0.03). Post-hoc analysis of this interaction revealed that the only condition with a significantly reduced amplitude was the unilateral scotoma, with a radius of 10° in the no defocus condition (estimate: 0.09°, standard error: 0.04°, *p* = 0.03).

**Table 1:** Linear mixed model for the no defocus ±1.5° disparity steps difference in vergence amplitude in experiment 2, with scotoma radius (continuous), scotoma format (categorical) and their interaction as fixed factors, and participant included as a random intercept.

*Effect of scotoma format and radius*	Estimate	Standard error	*p* value
Baseline (°) (bilateral 0°)	0.66	0.08	<0.001**
Scotoma Radius (degrees amplitude/degrees radius)	−0.01	0.005	0.03*
Scotoma Format (degrees amplitude from bilateral to unilateral)	0.07	0.05	0.14
Scotoma radius: scotoma format	−0.02	0.007	0.03*

The baseline model had a 0° scotoma radius and bilateral scotoma format, meaning no disruption of the images.

*p < 0.05 and **p < 0.001 for differences from baseline conditions.

### Experiment 2: Effect of Defocus Magnitude

The data were then modelled separately for the unilateral (Fig. 5) and bilateral (Fig. 6) defocus conditions, with defocus magnitude and scotoma radius as fixed continuous variables, scotoma format as a fixed categorical variable and participant as a random intercept. The interactions between defocus magnitude and scotoma radius, as well as defocus magnitude and scotoma format, were also included as fixed factors. In the unilateral defocus conditions, there was a significant decrease in vergence difference amplitude with an increase in both defocus magnitude and scotoma radius (Table 2, *p* < 0.001, for both). Post-hoc contrasts revealed that anisometropia ≥1.50 D led to responses that were reduced significantly from the no defocus condition for all scotoma radii (all *p* < 0.002). There was no statistically significant effect of scotoma format on vergence difference amplitude (Table 2, *p* = 0.81) in the presence of unilateral defocus. The interactions between defocus magnitude and scotoma radius, and defocus magnitude and scotoma format were not significant (Table 2, *p* > 0.21 for both).

**Table 2:** Unilateral and bilateral defocus magnitudes modelled separately with linear mixed models predicting the peak-to-trough vergence difference amplitude in experiment 2, with defocus magnitude (continuous), scotoma radius (continuous) and scotoma format (categorical) as fixed factors and participant as a random intercept.

Effect of defocus	Unilateral defocus			Bilateral defocus		
	Estimate	Standard error	*p* value	Estimate	Standard error	*p* value
Baseline (°) (0 D, 0° bilateral scotoma)	0.72	0.07	<0.001**	0.68	0.07	<0.001**
Defocus magnitude (° amplitude/D)	−0.08	0.02	<0.001**	−0.002	0.02	0.93
Scotoma radius (° amplitude/° radius)	−0.02	0.004	<0.001**	−0.02	0.005	<0.001**
Scotoma format (° amplitude from bilateral to unilateral)	−0.008	0.03	0.81	0.01	0.04	0.72
Defocus magnitude: scotoma radius	0.003	0.002	0.22	−0.003	0.003	0.18
Defocus magnitude: scotoma format	0.003	0.02	0.87	−0.01	0.02	0.50

The interactions between defocus magnitude and scotoma radius and defocus magnitude and scotoma format were included. The baseline model corresponds to the no defocus, 0° radius (no scotoma), bilateral scotoma condition, meaning no disruption of the images.

**p < 0.001 for differences from baseline conditions.

In bilateral defocus conditions, response amplitudes did not change significantly with an increase in the magnitude of defocus (Table 2, *p* = 0.93), whereas they did decrease significantly with an increase in scotoma radius (Table 2, *p* < 0.001). Once again, there was no significant effect of scotoma format. The interactions between defocus magnitude and scotoma radius and defocus magnitude and scotoma format were also not significant (Table 2, *p* > 0.18 for both). The responses in the control conditions for 2° and 8° scotoma radii indicated dissociation responses of the eyes of less than ±0.2° for these stimuli.

An important related question is how the final steady-state response amplitude is affected in these conditions, as it indicates the ultimate response accuracy that the participant is able to achieve. Open-loop amplitude was emphasised as the primary outcome measure because the scotoma was not stabilised on the retina throughout the trial, and therefore, more central regions of the retina would experience the images once eye movements were initiated. The steady-state amplitude figures and results presented in the Appendix only differ qualitatively from the open-loop results in unilateral defocus conditions, where participants were able to make more accurate eye movements in unilateral scotoma conditions than bilateral, and there was a borderline significant interaction between defocus magnitude and scotoma radius. Based on the assumption that retinal disparity cues would be limited to more peripheral retina if the scotomas were stabilised, the final steady-state accuracy of vergence responses of individuals with real scotomas would be predicted to be reduced more than was found here.

## Discussion

This study demonstrated the role of defocused peripheral retina in driving reflex vergence responses to maintain eye alignment, for large grayscale photographic images and step changes in stimulus disparity. The vergence response tuning functions for disparity were consistent with the previous literature, peaking at stimuli of 1.5°–2° and decreasing at greater disparities [23–25]. Even with the decreased open-loop vergence response in the presence of scotomas and defocus, the shape of the disparity tuning functions was retained up to a central scotoma radius of 25° and defocus values of 3 D (Figs. 3–6).

The open-loop vergence responses to these images decreased at bilateral and unilateral scotoma radii of 10° or greater in the first experiment, where scotoma radius was sampled as a categorical variable at values of 0°, 5°, 10°, 15°, 20° and 25°, and resulted in linear decreases with a radius of <10° in experiment 2. Although the vergence response decreased, participants were still able to respond in the correct direction without consistently dissociating to typical exophoric latent positions as represented in the group of participants who performed the control conditions in experiment 2. These results align with previous studies; Boman and Kertesz [38] and Kertesz and Hampton [11] also showed that binocular and monocular central scotomas that were stabilised on the retina resulted in reduced vergence responses at a diameter of ≥10°. Similarly, Eizenman et al. [12] demonstrated reduced open-loop vergence gain with an increase in tested monocular scotoma diameters of 2°–10°, with an increase in conjugate saccadic responses driven by the fellow eye without the scotoma. Also, Boman and Kertesz [38] observed that participants made asymmetrical vergence responses, more for unilateral than bilateral scotomas.

Although there was a decrease in the vergence response when stimuli were limited to the peripheral retina, none of the current participants with typical vision reported percepts of diplopia upon questioning after the experimental trials. This may be due to increases in Panum’s fusional area in the peripheral visual field [39–41], where a reduced vergence response may be sufficient to bring the target of interest into a percept of single vision. Previous studies suggested that vergence responses of individuals with suppression scotomas could be influenced by accommodative vergence [42, 43]. However, because this study only introduced a disparity cue, we can confidently conclude that these responses were driven solely by disparity-induced vergence, potentially in conflict with a stable defocus cue to the accommodation system. While blur-driven accommodation could increase the amplitude of the vergence response through accommodative convergence coupling, retinal disparity is the only cue capable of providing feedback about the accuracy of the vergence response.

No main effect of scotoma radius on response latency was found in experiment 1. However, the latencies did increase with increased log disparity magnitude, except for the 25° radius, indicating that larger disparities had an impact on temporal processing for small and moderate scotoma sizes. Latencies were shorter for convergent and divergent stimuli of <2° of disparity, with the only significant difference between convergence and divergence occurring for 0.5° of disparity (*p* < 0.001). This difference in latency between small convergence and divergence stimuli might result from the tendency to exophoria on dissociation in this group of participants. Takagi et al. [44] found that participants with exophoria had longer latency for convergence than divergence. The heterophorias of the participants in experiment 2, measured using the same experimental equipment, had a mean of 0.39° exophoria (range 1.17° exophoria to 0.37° esophoria).

The second experiment revealed no significant difference in vergence amplitudes between unilateral and bilateral scotomas for these natural scene images. These results align with the results of Boman and Kertesz [38], who reported comparable vergence responses with monocular and binocular scotomas of 10° and 15° in diameter. Their results indicated an asymmetry in vergence responses with monocular scotomas, and a symmetrical decrease in vergence response for binocular scotomas. The defocus results of the current study revealed that unilateral defocus of ≥1.50 D reduced vergence response amplitudes, whereas bilateral defocus did not. This extends the results of several previous studies, indicating that unilateral defocus is more detrimental than bilateral defocus [27–29]. This effect was similar for all of the radii tested (Figs. 5 and 6, Table 2) in both the unilateral and bilateral scotoma conditions. In combination, the results of the current study suggest a refractive correction of ≥1.50 D anisometropia may be important in maintaining eye alignment for patients with unilateral and bilateral scotomas. Future studies should determine whether unilateral defocus has a greater impact on the eye with or without a monocular scotoma. Here, data were collected with unilateral defocus presented to each eye in a balanced format.

This study expanded the understanding of the role of the peripheral retina in driving reflex vergence responses to grayscale photographic stimuli with natural scene statistics. A full understanding of motor fusion for natural stimuli will require future studies of dynamic three-dimensional scenes, with the potential for accommodative responses and interocular velocity differences to aid in the alignment response [45], and studies of participants with suppression and pathological scotomas to determine their ability to adapt to the demands of binocular function in their daily tasks. Investigations of the combined impact of scotomas and defocus on the developing visual system during infancy and early childhood are also of key importance if permanent experience-dependent vision loss for these children is to be prevented.

## Figures and Tables

**Fig. 7:**
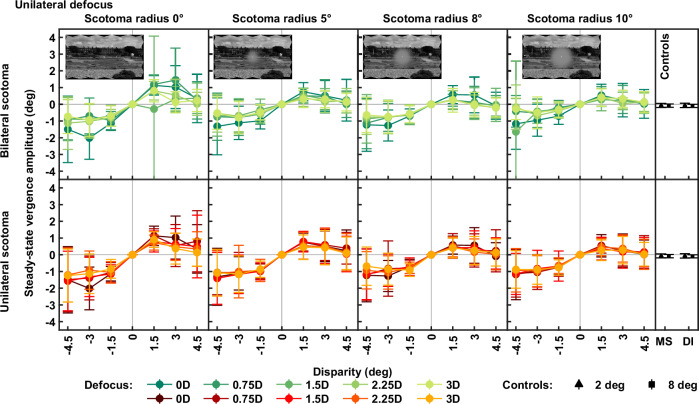
The effect of unilateral defocus magnitude and scotoma radius on steady-state vergence amplitude; Top row: bilateral scotoma, bottom row: unilateral scotoma. Positive values indicate crossed disparity and convergence. Deg degrees, MS monocular stimulus, DI different images.

**Fig. 8:**
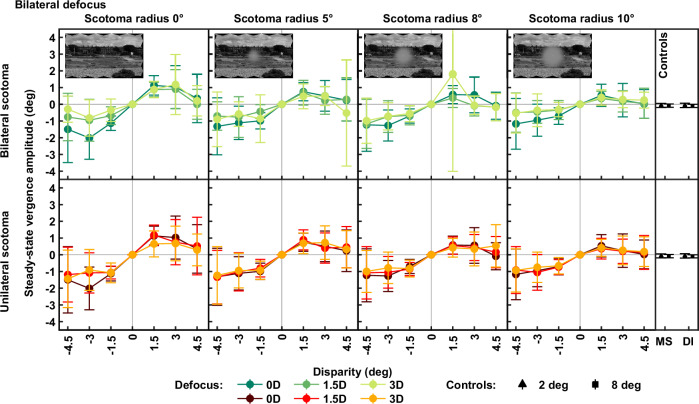
The effect of bilateral defocus magnitude and scotoma radius on steady-state vergence amplitude; Top row: bilateral scotoma, bottom row: unilateral scotoma. Positive values indicate crossed disparity and convergence. Deg degrees, MS monocular stimulus, DI different images.

## Data Availability

Data will be made available upon request.

## Appendix

**Table 3:** Unilateral and bilateral defocus magnitudes modelled separately with linear mixed models predicting the peak-to-trough difference in steady-state vergence response amplitudes for ±1.5° disparity stimuli in experiment 2.

Effect of defocus	Unilateral defocus			Bilateral defocus		
	Estimate	Standard error	*p* value	Estimate	Standard error	*p* value
Baseline (°) (0 D, 0° bilateral scotoma)	2.10	0.19	<0.001**	1.95	0.29	<0.001**
Defocus magnitude (° amplitude/D)	−0.22	0.07	0.002*	−0.11	0.13	0.39
Scotoma radius (° amplitude/° radius)	−0.13	0.02	<0.001**	−0.11	0.03	<0.001**
Scotoma format (° amplitude from bilateral to unilateral)	0.28	0.12	0.02*	0.36	0.24	0.13
Defocus magnitude: scotoma radius	0.02	0.009	0.03*	0.03	0.02	0.13
Defocus magnitude: scotoma format	0.01	0.07	0.87	−0.09	0.12	0.45

Defocus magnitude (continuous), scotoma radius (continuous) and scotoma format (categorical) were included as fixed factors with participant as a random intercept. The interactions between defocus magnitude and scotoma radius, and defocus magnitude and scotoma format were also included as fixed factors. The baseline model corresponds to the no defocus, 0° radius and bilateral scotoma conditions, meaning no disruption of the original image.

**p* < 0.05 and ***p* < 0.001 for differences from baseline conditions.
